# Health literacy among older adults 77+ in Sweden

**DOI:** 10.1093/eurpub/ckac131.331

**Published:** 2022-10-25

**Authors:** J Wångdahl, J Agerholm, A Liljas, J Heap, C Lennartsson

**Affiliations:** 1 Department of Public Health, Uppsala University, Uppsala, Sweden; 2 Aging Research Center, Karolinska Institutet and Stockholm University, Stockholm, Sweden; 3 Department of Global Public Health, Karolinska Institutet, Stockholm, Sweden; 4 Swedish Institute for Social Research, Stockholm University, Stockholm, Sweden

## Abstract

**Background:**

Health literacy (HL) is an essential component of an individual’s ability to gain access to, understand and use information to improve and maintain good health and is an important prerequisite for managing health and care needs. Therefore, knowledge on older people’s HL is vital to meet the care needs of the rapidly aging population efficiently and equitably. Knowledge on HL in the older population is limited, both in an international perspective and in Sweden. Challenges linked to limited HL are expected to increase with advanced age, emphasizing the importance of conducting a study using a nationally representative sample of older adults. The aim of the study was to explore the level of HL in older adults living in Sweden.

**Methods:**

A cross sectional study using data from the Swedish Panel Study of Living Conditions of the Oldest Old (SWEOLD), which is a nationally representative sample of the Swedish population aged 77+ years (N = 1500). SWEOLD covers areas such as health, socioeconomic and social factors. HL is assessed with the communicative and critical health literacy scale. The data was collected through telephone interviews in fall 2021 and spring 2022. Respondents not able to answer themselves were excluded.

**Results:**

Preliminary results show that 49 % of the participating adults aged 77+ have limited HL. HL was significantly associated with age (77-85 years OR: 1.80; 86+ OR: 3.04), education level (OR: 1.76), vision (OR: 2.40) and cognitive ability (OR: 0.88). HL was not significantly associated with sex.

**Conclusions:**

These preliminary results shows that a large proportion of Swedish older people might face difficulties related to accessing, understanding, appraising, and applying health information in ways which promote and maintain good health, and stresses the importance of examining the topic in greater detail.

**Key messages:**

• A high proportion of older adults in Sweden have limited health literacy.

• Health literacy among older adults in Sweden varies with age, level of education, vision and cognitive ability.

